# Mogroside V reduced the excessive endoplasmic reticulum stress and mitigated the Ulcerative colitis induced by dextran sulfate sodium in mice

**DOI:** 10.1186/s12967-024-05285-6

**Published:** 2024-05-21

**Authors:** Yue-Rong Tan, Si-Yang Shen, Xin-Yi Li, Peng-Fei Yi, Ben-Dong Fu, Lu-Yuan Peng

**Affiliations:** 1 College of Veterinary Medicine, Jilin University, No. 5333 Xi’an Road, Changchun, Jilin 130062 China; 2 State Key Laboratory for Diagnosis and Treatment of Severe Zoonotic Infectious Diseases, Jilin University, No. 5333 Xi’an Road, Changchun, Jilin 130062 China

**Keywords:** Mogroside V, Ulcerative colitis, Endoplasmic reticulum stress, Apoptosis

## Abstract

Ulcerative colitis (UC) is an idiopathic, chronic inflammatory condition of the colon, characterized by repeated attacks, a lack of effective treatment options, and significant physical and mental health complications for patients. The endoplasmic reticulum (ER) is a vital intracellular organelle in maintaining cellular homeostasis. Endoplasmic reticulum stress (ERS) is induced when the body is exposed to adverse external stimuli. Numerous studies have shown that ERS-induced apoptosis plays a vital role in the pathogenesis of UC. Mogroside V (MV), an active ingredient of Monk fruit, has demonstrated excellent anti-inflammatory and antioxidant effects. In this study, we investigated the therapeutic effects of MV on dextran sulfate sodium (DSS)-induced UC and its potential mechanisms based on ERS. The results showed that MV exerted a protective effect against DSS-induced UC in mice as reflected by reduced DAI scores, increased colon length, reduced histological scores of the colon, and levels of pro-inflammatory cytokines, as well as decreased intestinal permeability. In addition, the expression of ERS pathway including BIP, PERK, eIF2α, ATF4, CHOP, as well as the apoptosis-related protein including Caspase-12, Bcl-2 and Bax, was found to be elevated in UC. However, MV treatment significantly inhibited the UC and reversed the expression of inflammation signaling pathway including ERS and ERS-induced apoptosis. Additionally, the addition of tunicamycin (Tm), an ERS activator, significantly weakened the therapeutic effect of MV on UC in mice. These findings suggest that MV may be a therapeutic agent for the treatment of DSS-induced UC by inhibiting the activation of the ERS-apoptosis pathway, and may provide a novel avenue for the treatment of UC.

## Introduction

Ulcerative colitis (UC) is an idiopathic, chronic inflammatory disorder, which starts distally and can gradually extend proximally through the whole colon [1]. Clinical manifestations typically include bloody diarrhoea, frequency, abdominal pain, fatigue and fecal incontinence, which is increasing in incidence and prevalence [2]. Although UC was originally prevalent in North America and Europe, its incidence has been gradually increasing in developing country such as China in recent years [3], and it tends to affect young adults, imposing a heavy societal burden [4]. From initial use of traditional anti-inflammatory drugs, such as sodium aminosalicylate [5] and corticosteroids, to the introduction of biologic agents, such as immunosuppressants, anti-tumor necrosis factor alpha, and anti-IL-17 classes [6], there has been a growing understanding of UC treatment. However, issues such as hormone dependence, adverse reactions, and drug resistance to these treatments persist, and the objective of developing a safe and effective treatment remains a significant challenge [7]. Furthermore, the high cost of treatment remains unresolved. Consequently, it is imperative to identify a novel therapeutic strategy.

The intestinal barrier serves as an important protective barrier against external pathogens and harmful substances [8]. Although the pathogenesis of UC is not yet fully understood, the occurrence of UC appears to be related to disruption in the intestinal mucosa barriers and resultant perturbations of the gut microbiota [9]. When the intestinal barrier is compromised, intestinal permeability increases, and exogenous toxins or bacteria can more easily enter the body, leading to the development of UC [10]. The endoplasmic reticulum (ER) functions as a quality-control organelle for protein homeostasis, or “proteostasis”. The accumulation of misfolded proteins in the ER leads to endoplasmic reticulum stress (ERS), resulting in activation of the unfolded protein response (UPR) that aims to restore protein homeostasis [11]. ERS and defects in UPR signaling are emerging as key contributors to a growing list of human diseases, including diabetes, liver diseases, neurodegeneration, and cancer [12]. Several studies have shown that the ERS pathway plays a crucial role in maintaining the integrity of the intestinal mucosal barrier [13]. Excessive ERS signaling or persistent UPR activation can lead to apoptotic pathways [14], intestinal epithelial cell death, abnormal secretion of intestinal mucosal barrier proteins, and activation of pro-inflammatory responses in the intestine, leading to UC [15]. Modulation of ERS has emerged as a new therapeutic strategy in the treatment of UC.

Traditional Chinese medicine (TCM) is used as complementary and alternative therapies all over the world for the treatment of various diseases. Due to its multi-targeted mode of action and few side effects, it has unique advantages for treating chronic diseases, including definite curative effects, effective maintenance, recurrence rate reduction, and only minor toxic side effects [16]. With the continuous development of theoretical research in TCM, TCM has shown great progress in the research of treating UC [17]. Mogroside V (MV) is a sweet chemical extracted from the medicinal and food source of Siraitia grosvenorii, and has been used to prepare specially flavoured foods [18]. It has demonstrated pharmacological effects such as antioxidant and anti-tumour properties [19], and is commonly used as a cough suppressant and lung humectant [20]. Previous studies have shown that mogroside V can alleviate oxidative damage [21]. However, the efficacy of MV in treating UC remains unclear. Therefore, this study aimed to investigate the therapeutic effects of MV on dextran sulfate sodium-induced UC and explore its underlying mechanisms based on the ERS, to provide potential therapeutic drugs for the prevention and treatment of UC.

## Materials and methods

### Materials

The MV (purity > 99%) was purchased from Chengdu Must Biotechnology Co., Ltd. (Chengdu, China). Female C57BL/6J mice were obtained from the Experimental Animal Centre of Baiqiu’en School of Medicine, Jilin University (Jilin, China).

### Animals and experimental design

Female C57BL/6J mice weighing between 20 and 22 g were housed in a temperature-controlled room at 24 ± 2 °C with a 12-hour light/dark cycle. The mice were allowed to acclimatize for one week before grouping based on similar body weight for the experiment. All animal procedures were conducted following the provisions of the Guide for the Care and Use of Laboratory Animals of Jilin University and were approved by the Animal Ethics Committee of Jilin University. All procedures requiring anesthesia were performed under isoflurane.

To determine the therapeutic effect of MV for treatment of UC, female C57BL/6J mice were randomly divided into five groups (*n* = 6) including control group, model group and DSS + MV (25, 50, 100 mg/kg) groups. Except for the control group, the mice in the other groups drank 3% DSS freely for 7 days according to the previous report [22]. Meanwhile, mice in the control and model groups were orally administered deionized water. Mice in the DSS + MV groups were orally administered MV dissolved in deionized water (25, 50 and 100 mg/kg). (1) control group: normal drinking water plus saline by gavage; (2) model group: 3% DSS (molecular weight: 36,000–50,000, MP Biomedical, Morgan Irvine, CA, USA) added to drinking water and saline by gavage; (3) DSS low-dose MV: 3% DSS was added to the mice’s drinking water and an equal amount of low-concentration MV (25 mg/kg BW) was added by gavage; (4) DSS medium-dose MV: 3% DSS was added to the drinking water of mice and an equal amount of medium-concentration MV (50 mg/kg BW) was administered by gavage; (5) DSS high-dose MV: Mice were given 3% DSS in their drinking water and an equal amount of high-concentration MV (100 mg/kg BW) by gavage.

To investigate whether MV exerts its therapeutic effect on murine colitis through the ERS pathway, female C57BL/6J mice were divided into five groups, each consisting of six mice. The groups were as follows: (1) control group: normal drinking water plus saline by gavage; (2) model group: 3% DSS added to drinking water and saline by gavage; (3) DSS high-dose MV group: 3% DSS was added to mice drinking water, and an equal amount of high-concentration MV (100 mg/kg BW) was administered by gavage; (4) DSS high-dose MV and 4-PBA group: 3% DSS was added to the drinking water of mice, and an equal amount of high-concentration MV (100 mg/kg BW) and 4-phenylbutyric acid (4-PBA, MCE USA, 500 mg/kg BW) were administered by gavage; (5) DSS high-dose MV and Tm group: Mice were given 3% DSS in their drinking water and equal amounts of high concentrations of MV (100 mg/kg BW) and tunicamycin (Tm, Aladdin, Shanghai, China, 1 mg/kg BW) by gavage.

### DAI and histopathological inflammation score

The Disease Activity Index (DAI) is a scoring system that evaluates the severity of colitis by assessing weight loss, stool texture, and stool bleeding. The scoring for each aspect is as follows: weight loss rate (0: none, 1: 1–5%, 2: 6–10%, 3: 11–20%, 4: >20%); stool texture (0: normal, 1: loose, 2: semi-formed, 3: loose stool, 4: watery diarrhea); and stool bleeding (0: no bleeding, 1: positive occult blood, 2: blood in stool, 3: perianal bleeding, 4: bloody diarrhea) [23].

The pathological histological score is based on the following three aspects: the degree of epithelial damage and ulcer formation, the extent of ulcer depth, and the degree of inflammatory cell infiltration. The scoring details for each aspect are as follows: degree of injury and ulceration (0: none, 1: slight erosion, 2: diffuse erosion, 3: ulceration); depth of ulceration (0: none, 1: submucosal, 2: muscular layer, 3: plasma layer); degree of inflammatory cell infiltration (0: none, 1: mild, confined to the mucosa, 2: moderate, reaching the muscular layer, 3: severe, reaching the plasma layer) [24].

### Intestinal permeability assay

Intestinal permeability in mice was measured using non-absorbable FITC-dextran (4 kDa). Mice were fasted for 8 h prior to the experiment. FITC-dextran was prepared at a concentration of 50 mg/mL and administered by gavage at a dose of 0.1 mL/mouse. Blood samples were collected from the eyes 4 h later, and the supernatant was collected after centrifugation. A standard curve was generated by preparing a 20 µg/mL solution of FITC-dextran and successively diluting it in the following ratios: 10 µg/mL, 5 µg/mL, 2.5 µg/mL, 1.25 µg/mL, 0.625 µg/mL, and 0.3125 µg/mL. The samples to be tested were diluted with PBS (1/2 dilution) and 100 µL of the solution was added to each well. The absorbance was measured using an excitation wavelength of 480 nm and a measuring wavelength of 520 nm. The standard curve was plotted, and the concentration of the samples was converted according to the standard curve.

### RNA extraction and qRT-PCR

Total RNA was extracted from the samples using Trizol reagent (PolymerMei, China) following the manufacturer’s instructions. Subsequently, cDNA was synthesized using a reverse transcription kit (PolymerMei, China). qRT-PCR was performed using specific primers, the sequences of which are provided in Table 1.

**Table 1:** The primer sequence in this study

Primer name	Sequence (5’ to 3’)
Mouse-ZO-1-F	GAGCAGGCTTTGGAGGAGAC
Mouse-ZO-1-R	TGGGACAAAAGTCCGGGAAG
Mouse-Occludin-F	TTTCAGGTGAATGGGTCACCG
Mouse-Occludin-R	GAGCAAAATGTCCAGGCTCC
Mouse-Claudin-1-F	CCACCATTGGCATGAAGTGC
Mouse-Claudin-1-R	CCCAATGACAGCCATCCACA
Mouse-BIP-F	TGTGTGTGAGACCAGAACCG
Mouse-BIP-R	GCAGTCAGGCAGGAGTCTTA
Mouse-PERK-F	CGCGTCGGAGACAGTGTTT
Mouse-PERK-R	GTCCTCCACGGTCACTTCG
Mouse-eIF2α-F	ATGCCGGGGCTAAGTTGTAGA
Mouse-eIF2α-R	AACGGATACGTCGTCTGGATA
Mouse-ATF4-F	CCTGAACAGCGAAGTGTTGG
Mouse-ATF4-R	TGGAGAACCCATGAGGTTTCAA
Mouse-CHOP-F	AAGCCTGGTATGAGGATCTGC
Mouse-CHOP-R	TTCCTGGGGATGAGATATAGGTG
Mouse-Bax-F	AGACAGGGGCCTTTTTGCTAC
Mouse-Bax-R	AATTCGCCGGAGACACTCG
Mouse-Bcl-2-F	GCTACCGTCGTGACTTCGC
Mouse-Bcl-2-R	CCCCACCGAACTCAAAGAAGG
Mouse-Caspase-12-F	TAGGGGAAAGTGCGAGTTTCA
Mouse-Caspase-12-R	GGGCCAATCCAGCATTTACCT

### Histological analysis

To investigate the pathological changes in mouse colon tissue, 4% PFA-fixed paraffin-embedded colon tissue sections were prepared and subjected to hematoxylin-eosin (H&E) staining and Alcian blue-Schiff’s periodate (AB-PAS) staining. Positive staining for adhesion proteins was quantitatively analyzed using IPWIN60. Immunohistochemical staining for anti-ZO-1 (from Sevilla, Wuhan, China) was also performed on mouse colon tissues.

### Western blot analysis

Proteins were extracted from mouse colon tissues through homogenization and lysis using pre-cooled RIPA lysis buffer. Protein content was determined using the BCA protein assay kit (Thermo Fisher, USA). Protein samples containing 30 µg of total protein were collected, separated by SDS-PAGE, and transferred to PVDF membranes (Thermo Fisher, USA). The membranes were blocked with 5% BSA for 4 h at room temperature, followed by incubation at 4 °C with specific primary antibodies including ZO-1 (1:1000), Occludin (1:2000), Claudin-1 (1:1000), BIP (1:1000), PERK (1:1000), ATF4 (1: 1000), eIF2α (1:1000), CHOP (1:1000), Bcl-2 (1:1000), Bax (1:1000), Caspase-12 (1:1000), and GAPDH (1:1000). The membranes were then incubated with HRP-conjugated secondary antibodies (1:5000) for 2 h at room temperature. Finally, spots were visualized using an enhanced chemiluminescence immunoblotting detector (Mona, USA) and quantified using ImageJ software (National Institute of Mental Health, USA).

### Data analysis

Data analysis was performed using the mean and standard deviation. Differences between the two groups were determined using a T-test with SPSS 17.0 statistical software (Chicago, IL, USA). Differences between groups were also analyzed using one-way ANOVA or two-way ANOVA with GraphPad Prism 8 (GraphPad Software Inc., La Jolla, CA, USA). Statistical significance was defined as *P* < 0.05.

## Results

### MV treatment alleviated the severity of UC in mice

**Fig. 1:**
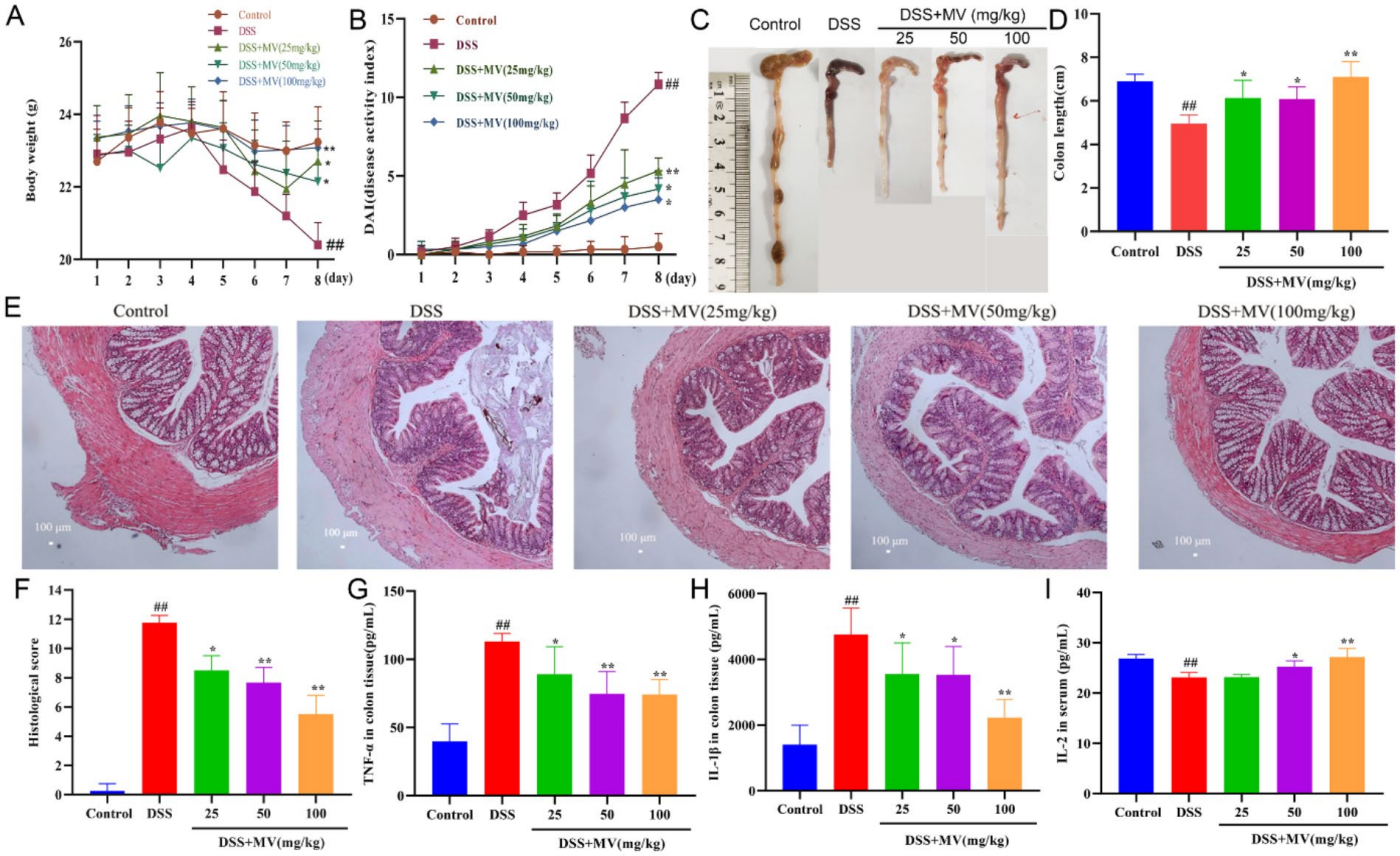
Effect of MV on the severity of UC mice. (**A**) the changes of body weight. (**B**) DAI scores. (**C**) representative images of colons. (**D**) colon lengths. (**E**) histopathologic examination of colon. (**F**) histological score. The secretion of cytokines of (**G**) TNF-α, (**H**)IL-1β, (**I**) IL-2. Values are expressed as mean ± SEM. ^#^*p* and ^##^*p* represents a significant difference compared with the control group. ^*^p and ^**^p represent a significant difference compared with the DSS group

The recordings of the mice’s weight loss showed that the mice on the DSS-containing diet began losing weight on day 4, and the weight loss increased over time. However, MV treatment obviously slowed the DSS-induced weight loss in the mice (Fig. 1A). Mice in the MV-treated group had lower DAI scores compared to the model group, with a more pronounced difference on day 7 (Fig. 1B). In the model group, the colon length was significantly shorter, swollen, and the contents were indeterminate and celiac-like, compared to the control group. However, MV treatment significantly reversed the shortening of the colon in a dose-dependent manner, and the colon length and faecal morphology in the MV group were similar to the control group (Fig. 1C and D). Histological analysis of colon in DSS group displayed extensive ulcerative damage, loss of glandular crypts, extensive loss of goblet cell, and infiltration of inflammatory cells. In contrast, an increased number of goblet cells, a general recovery of crypt glands, and a small amount of inflammatory infiltration were seen in the colon sections of the mice after MV treatment (Fig. 1E and F). Furthermore, MV treatment significantly reduced the secretion of proinflammatory cytokine IL-1β and TNF-α, improved the release of anti-inflammatory cytokine IL-2 in colonic tissues induced by DSS (Fig. 1G-I).

### MV repaired the intestinal barrier in UC mice

**Fig. 2:**
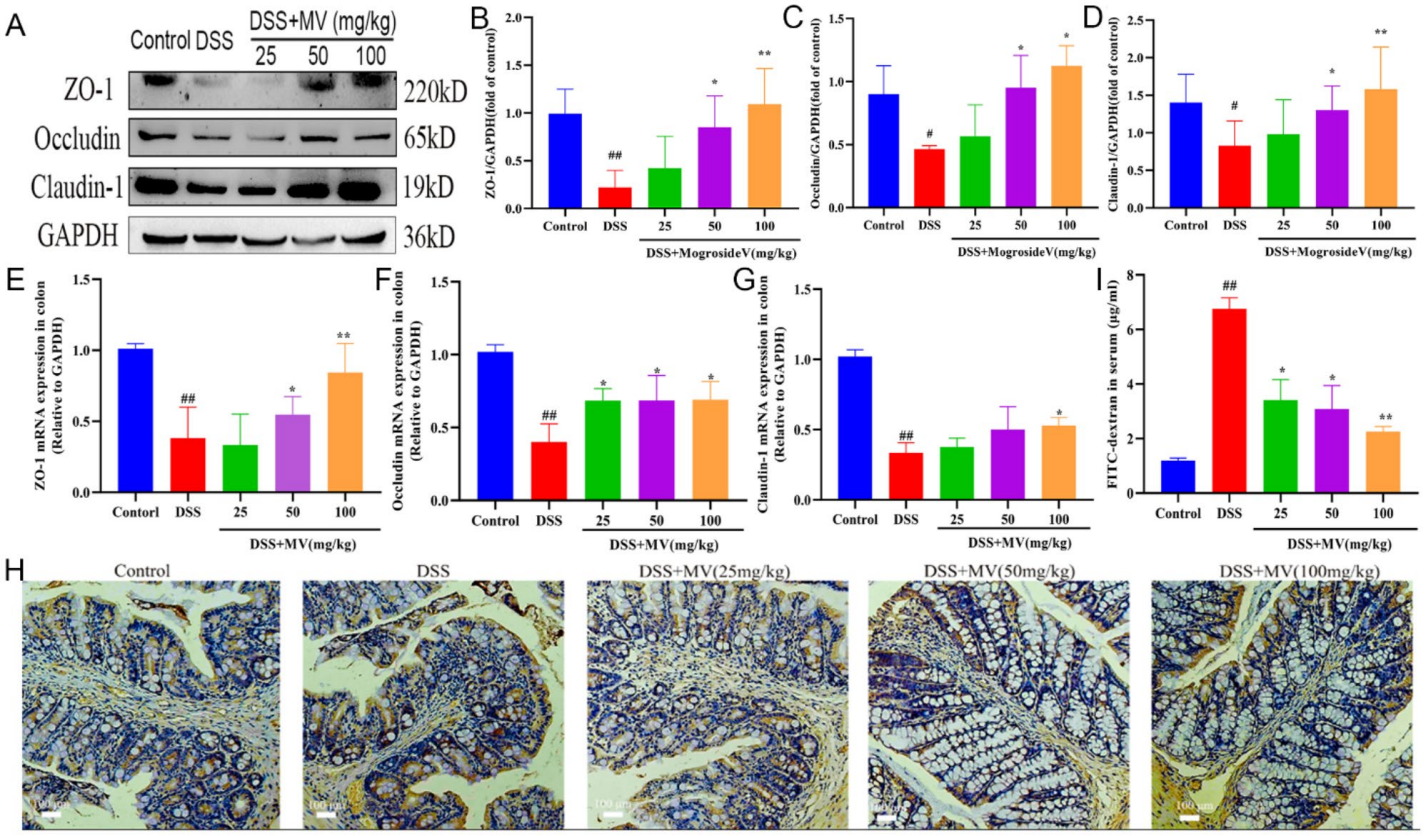
Effect of MV on the intestinal permeability. (**A**) representative western blotting of ZO-1, Occludin, Claudin-1. GAPDH was determined as the loading control. Protein expression quantification of (**B**) ZO-1, (**C**) Occludin and (**D**) Claudin-1. The relative mRNA expression of (**E**) ZO-1, (**F**) Occludin and (**G**) Claudin-1. (**H**) Immunohistochemical staining results of ZO-1. (**I**) the serum FITC level of mice. Values are expressed as mean ± SEM. ^#^*p* and ^##^*p* represents a significant difference compared with the control group. ^*^p and ^**^p represent a significant difference compared with the DSS group

Intestinal permeability is a crucial indicator of the response to intestinal mucosal injury and the assessment of intestinal barrier function. In this study, we estimated the expression of tight junction proteins ZO-1, Occludin, and Claudin-1 was detected by western blot and qRT-PCR, the results showed that the expression of ZO-1, Occludin, and Claudin-1 was significantly reduced in the DSS group compared with the control group. After MV treatment, its expression level was significantly restored (Fig. 2A-G). Moreover, the immunohistochemical staining showed that the expression of ZO-1 in goblet cells was significantly reduced in the DSS group, while the expression of ZO-1 was enhanced with the increase of MV treatment concentration (Fig. 2H). In addition, intestinal permeability by measuring serum FITC-dextran levels. The serum FITC-dextran level of mice in the model group was significantly higher than that of the blank group. However, MV treatment effectively reduced level of serum FITC-dextran (Fig. 2I).

### MV inhibited the ERS-UPR signal pathway activation in UC mice

**Fig. 3:**
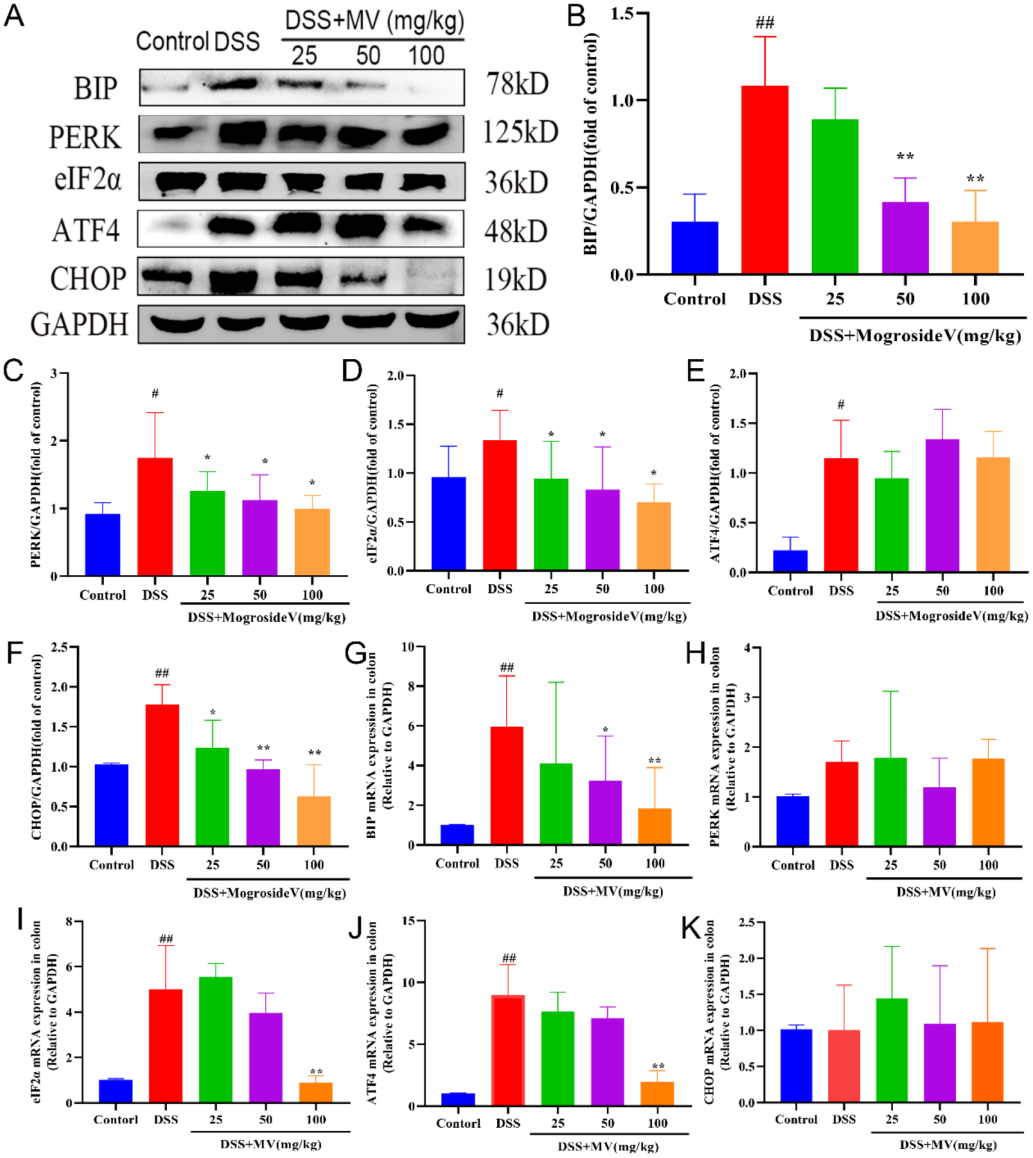
Effect of MV on the ERS-UPR signal pathway. (**A**) representative western blotting of ERS-UPR signal pathway. GAPDH was determined as the loading control. Protein expression quantification of (**B**) BIP, (**C**) PERK, (**D**) eIF2α, (**E**) ATF4, (**F**) CHOP. And the relative mRNA expression of (**G**) BIP, (**H**) PERK, (**I**) eIF2α, (**J**) ATF4, (**K**) CHOP. Values are expressed as mean ± SEM. ^#^*p* and ^##^*p* represents a significant difference compared with the control group. ^*^p and ^**^p represent a significant difference compared with the DSS group

ERS is the result of the accumulation of unfolded and misfolded proteins in the ER. The UPR acts as a highly conserved signaling mechanism that enables cells to respond to and calm ERS. In this study, we investigated the protein and mRNA levels of ERS -UPR signal pathway to elevate the regulatory effect of MV on ERS by western blot and qRT-PCR. The results showed that DSS treatment significantly increased the protein and mRNA expression of ERS-UPR signal pathway including BIP, PERK, eIF2α, ATF4 and CHOP when compared to control group. However, administration of MV obviously inhibited the expression of ERS-UPR signal pathway especially in protein levels (Fig. 3).

### MV inhibited the ERS-associated apoptosis in UC mice

**Fig. 4:**
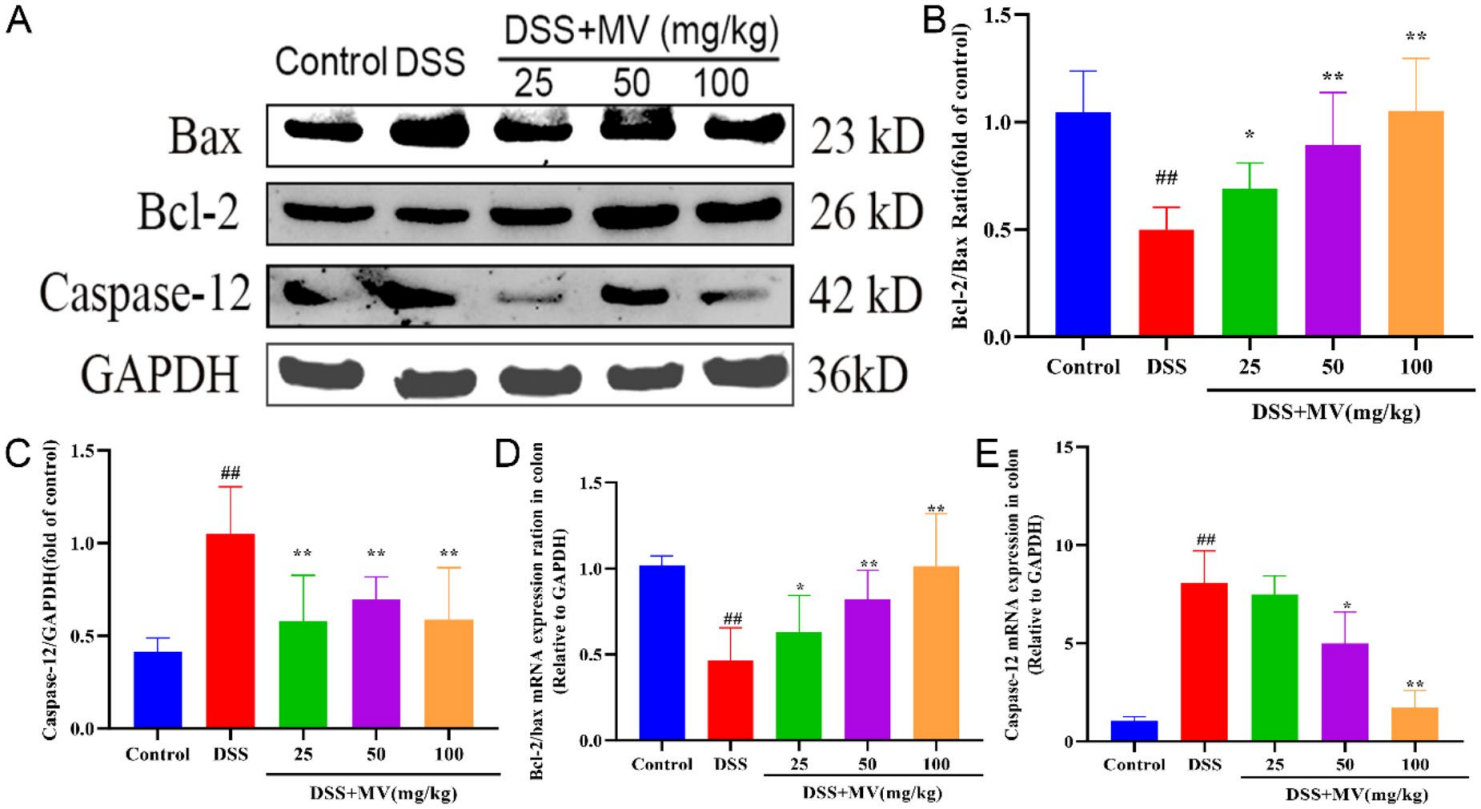
Effect of MV on the ERS-associated apoptosis pathway. (**A**) representative western blotting of Bax, Bcl-2 and Caspase-12. GAPDH was determined as the loading control. Protein expression quantification of (**B**) Bcl-2/Bax ratio, (**C**) Caspase-12. And the relative mRNA expression of (**D**) Bcl-2/Bax ratio, (**E**) Caspase-12. Values are expressed as mean ± SEM. ^#^*p* and ^##^*p* represent a significant difference compared with the control group. ^*^p and^**^p represent a significant difference compared with the DSS group

Prolonged ERS can activate chop proteins in the UPR process, leading to ERS-associated apoptosis and the onset of mitochondria-dependent pathway apoptosis. To examine the effects of MV on ERS-induced apoptosis, we investigated the changes in the ratio of the ERS-specific apoptotic protein Caspase-12 and apoptosis-regulating protein family Bcl-2 and Bax by qRT-PCR and western blot. The results showed that the expression of Caspase-12 was significantly increased and the ratio of Bcl-2/Bax was significantly reduced in DSS group when compared to the control group. However, MV treatment significantly inhibited the expression of Caspase-12 and improved the ratio of Bcl-2/Bax to restrain the ERS-induced apoptosis (Fig. 4).

### Treatment with Tm reversed the effect of MV on DSS-induced ERS-UPR signal pathway in UC mice

**Fig. 5:**
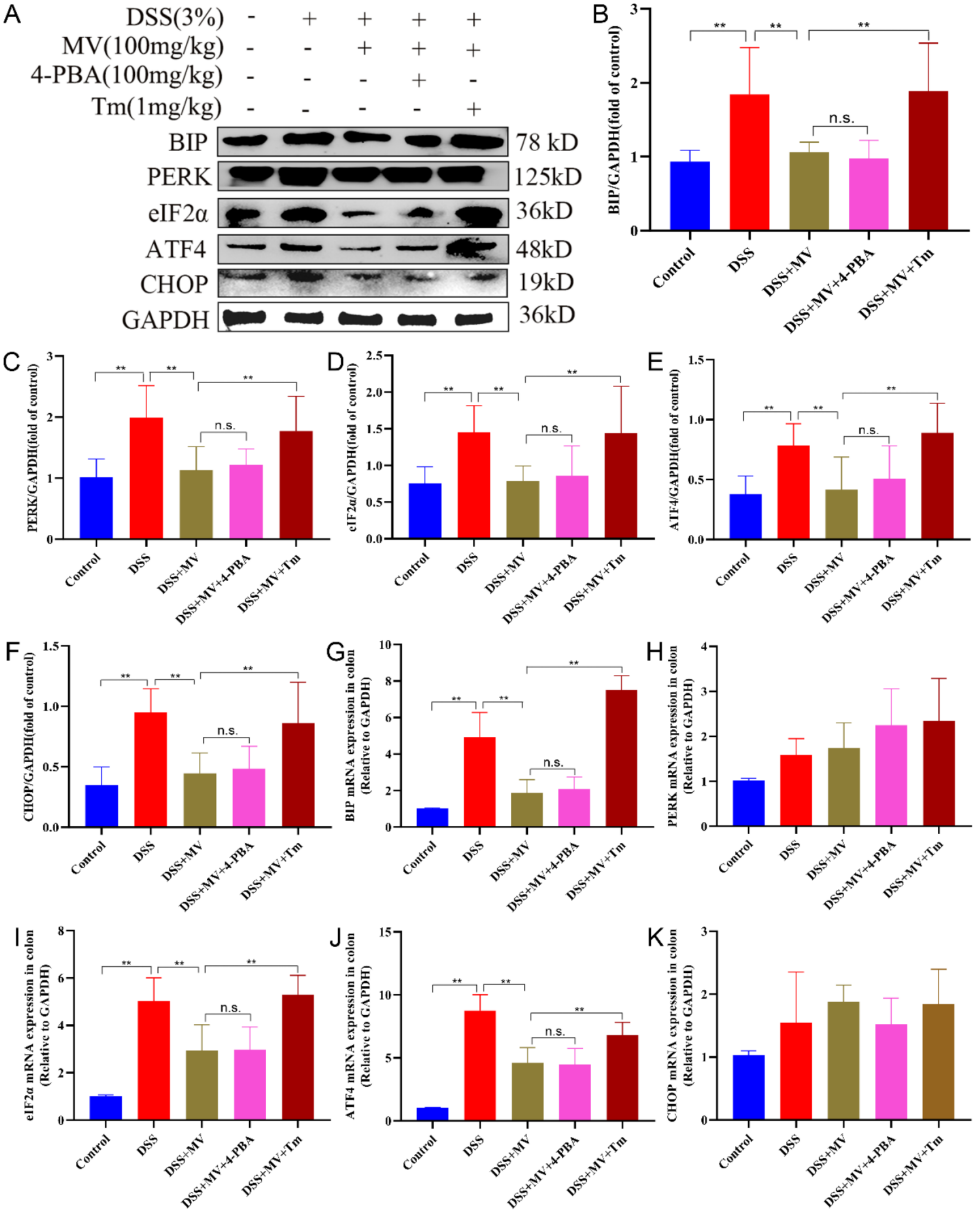
Effect of 4-PBA and Tm on the MV inhibiting ERS. (**A**) representative western blotting of ERS-UPR signal pathway. GAPDH was determined as the loading control. Protein expression quantification of (**B**) BIP, (**C**) PERK, (**D**) eIF2α, (**E**) ATF4, (**F**) CHOP. And the relative mRNA expression of (**G**) BIP, (**H**) PERK, (**I**) eIF2α, (**J**) ATF4, (**K**) CHOP. Values are expressed as mean ± SEM. n.s. was considered to have no significant difference. ^*^p < 0.05 and ^**^p < 0.01 represent a significant difference

To investigate whether the therapeutical effect of MV on UC was related to the ERS pathway, we administered ERS inhibitor, 4-PBA, and activator, Tm, to DSS mice at high doses of MV and observed their effects on UC mice. The results displayed that treatment with MV significantly inhibited the expression of BIP, PERK, eIF2α, ATF4 and CHOP induced by DSS. However, inhibition of ERS by Tm significantly weakened the inhibiting effect of MV on ERS-UPR pathway. In contrast, the expression of BIP, PERK, eIF2α, ATF4 and CHOP in the colonic tissue of DSS + MV + 4-PBA group was showing no significant difference compared to DSS + MV group. These results suggested that inhibition of ERS alleviate the inhibiting effect of MV on ERS-UPR signal pathway (Fig. 5).

### Activation of ERS reversed the effect of MV on DSS-induced ERS-associated apoptotic in UC mice

**Fig. 6:**
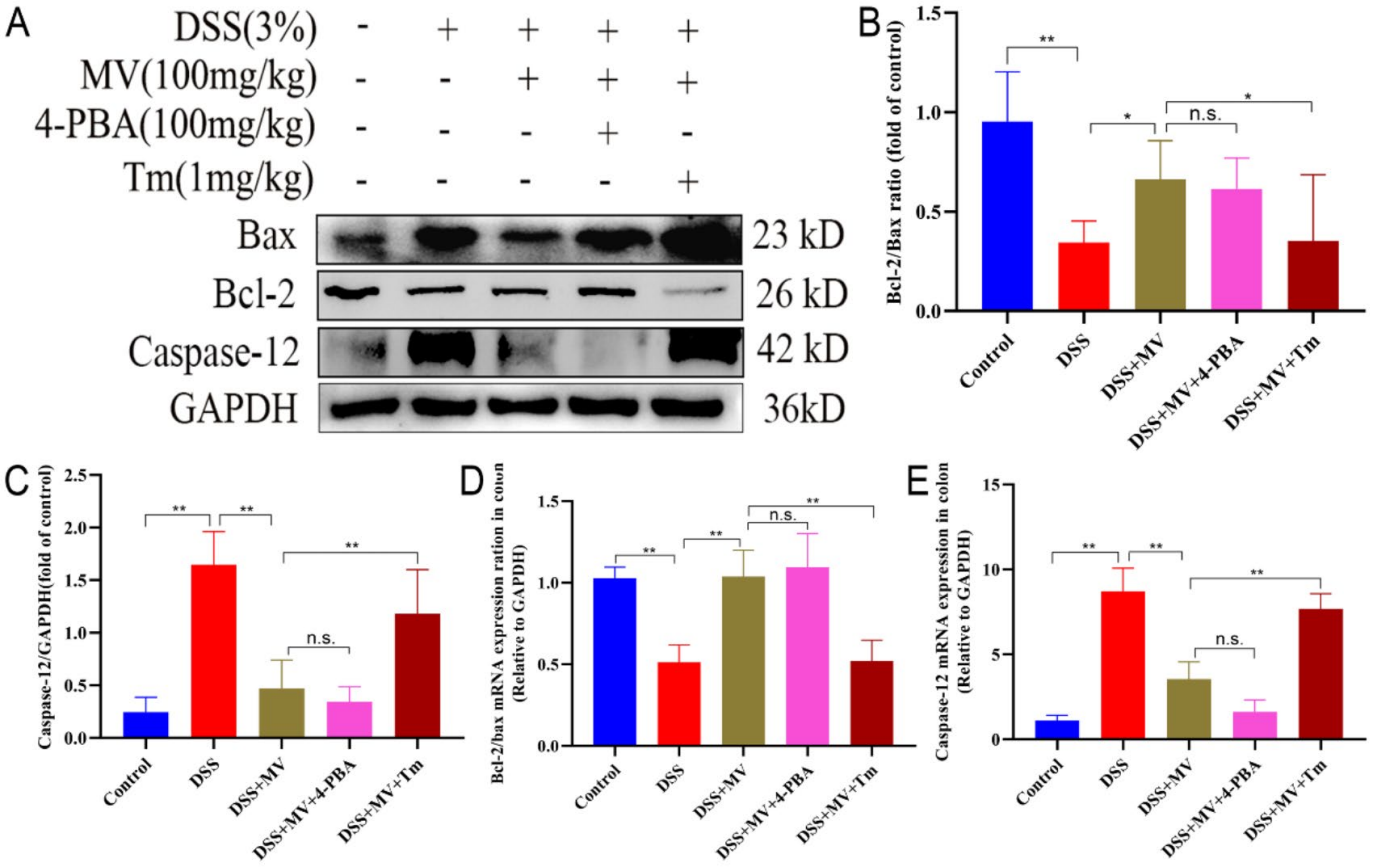
Effect of 4-PBA and Tm on the MV restraining ERS-related apoptosis. (**A**) representative western blotting of Bax, Bcl-2 and Caspase-12. GAPDH was determined as the loading control. Protein expression quantification of (**B**) Bcl-2/Bax ratio, (**C**) Caspase-12. And the relative mRNA expression of (**D**) Bcl-2/Bax ratio, (**E**) Caspase-12. Values are expressed as mean ± SEM. n.s. was considered to have no significant difference. ^*^p < 0.05 and ^**^p < 0.01 represent a significant difference

We further investigated the effects of inhibition and activation of ERS by 4-PBA and Tm on apoptotic proteins associated with the MV treatment group. Compared to the DSS + MV group, the DSS + MV + Tm group was observed to reverse the decrease in Caspase-12 protein expression, eliminate the therapeutic effect of MV, and further upregulate the Bcl-2/Bax ratio. Meanwhile, 4-PBA induced ERS inhibition had no significant effect on the regulatory effect of MV on ERS associated apoptosis. These results suggest that inhibition of ERS reversed the effect of MV on DSS-induced ERS-associated apoptotic in UC mice (Fig. 6).

### Activation of ERS reversed the effect of MV on the severity of UC

**Fig. 7:**
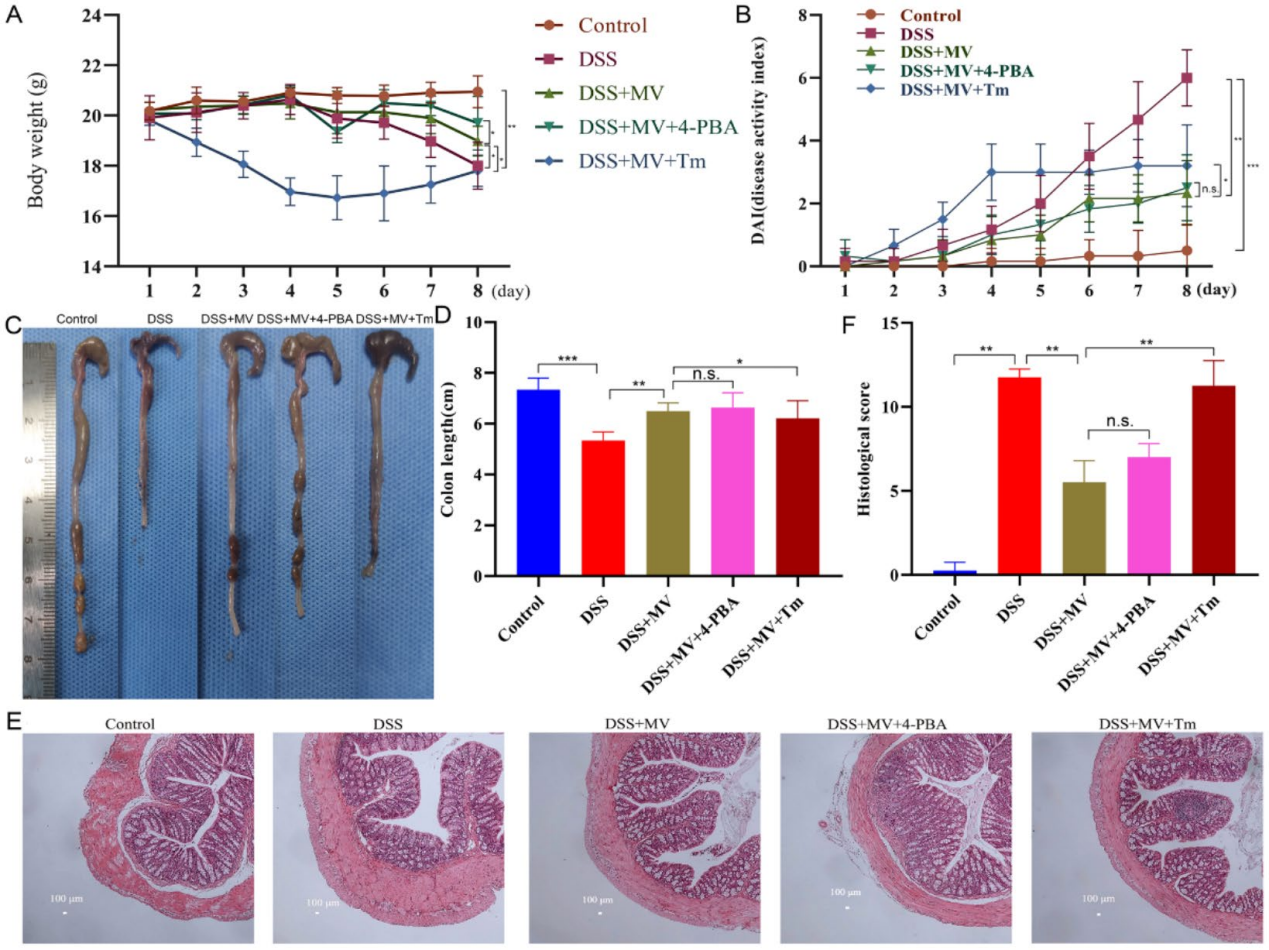
Effect of 4-PBA and Tm on the MV reducing the severity of UC mice. (**A**) the changes of body weight. (**B**) DAI scores. (**C**) representative images of colons. (**D**) colon lengths. (**E**) histopathologic examination of colon. (**F**) histological score. Values are expressed as mean ± SEM. n.s. was considered to have no significant difference. ^*^p < 0.05 and ^**^p < 0.01 represent a significant difference

The results showed that the addition of Tm counteracted the therapeutic effect of MV, with a similar degree of weight loss as the model group and a relatively higher DAI score, the shorter colon length and heavier histopathological damage than MV group. On the other hand, the application of 4-PBA had no obvious influence on the MV alleviating the severity of UC (Fig. 7).

### Activation of ERS reversed the effect of MV on the intestinal barrier

**Fig. 8:**
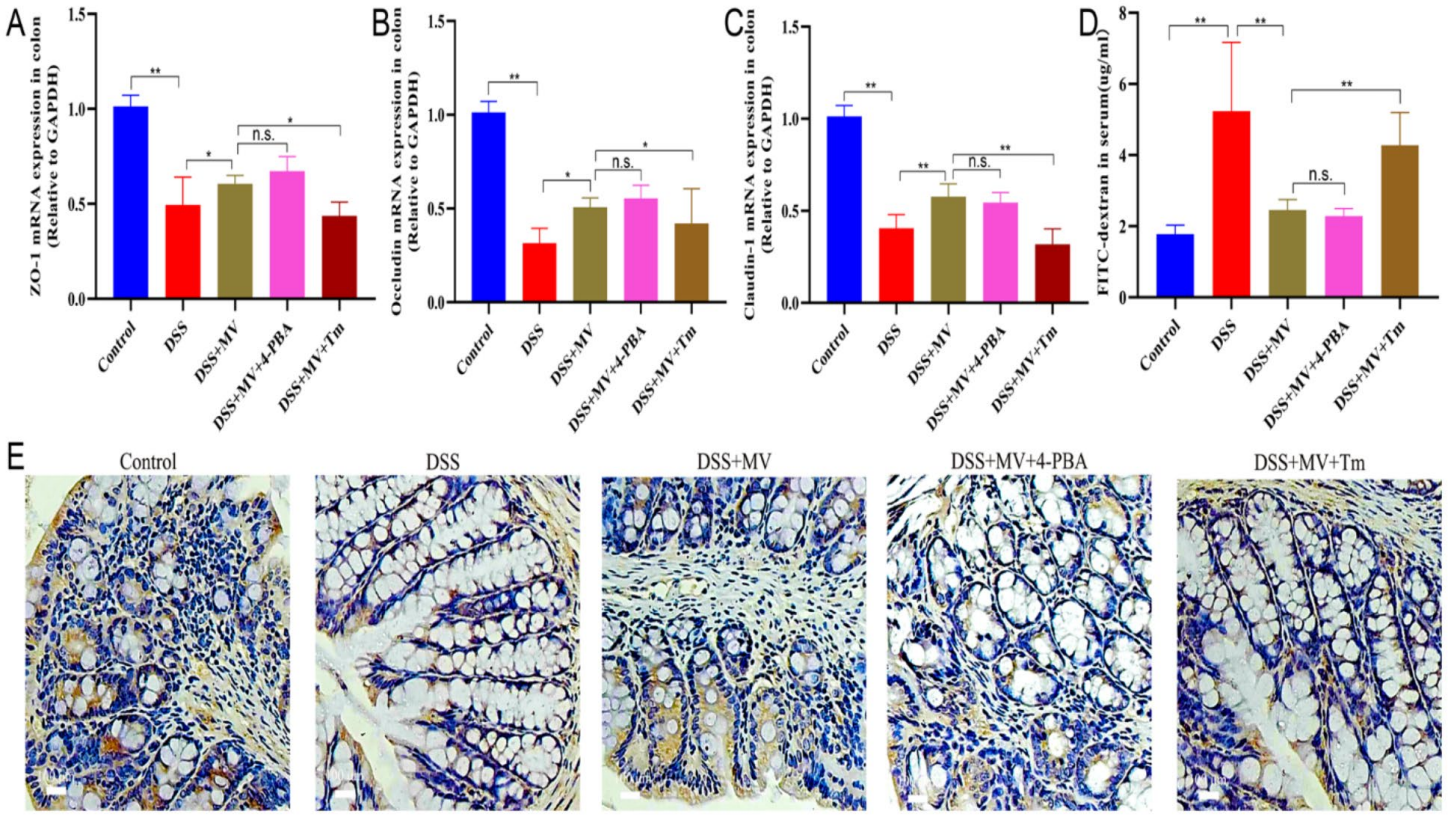
Effect of 4-PBA and Tm on the MV improving the intestinal barrier of UC mice. The relative mRNA expression of (**A**) ZO-1, (**B**) Occludin and (**C**) Claudin-1. (**D**) the serum FITC level of mice. (**E**) Immunohistochemical staining results of ZO-1. Values are expressed as mean ± SEM. n.s. was considered to have no significant difference. ^*^p < 0.05 and ^**^p < 0.01 represent a significant difference

Results of the intestinal permeability test indicated that Tm treatment significantly weakened the effects of repairing the intestinal barrier of MV showing by increased serum FITC-dextran levels, reduced expression of ZO-1, Occludin and Claudin-1. Conversely, the 4-PBA treatment had no significant difference from DSS + MV group (Fig. 8).

## Discussion

UC is a chronic, nonspecific inflammatory bowel disease that has been classified by the World Health Organization as a modern refractory disease. It has a long course and is challenging to cure. Recent years have witnessed a growing trend of applying TCM to UC owing to their low toxicity and side-effects relative to western medicine [16]. In this study, MV, a natural pharmaceutical monomer with anti-inflammatory and antioxidant effects, was found to exhibit good therapeutic effects at concentrations ranging from 25 to 100 mg/kg, which significantly increased with increasing concentrations. Inflammatory factors can cause damage to intestinal epithelial cells, increased intestinal permeability, and inflammatory cell infiltration, and their levels are significantly elevated during the onset and progression of colitis [25, 26]. Treatment with MV significantly reduced the releases of proinflammatory factors TNF-α, IL-1β, and increased the levels of anti-inflammatory factors IL-2 in mice with colitis, indicating its good anti-inflammatory effects. Moreover, the expression of tight junction protein ZO-1, Claudin-1, and Occludin in intestinal mucosal epithelial cells decreased, causing an increase in intestinal permeability [27], which leads to the entry of pathogenic microorganisms and harmful substances into the intestinal mucosa, activating immune cells to produce inflammatory responses and further aggravating the development of colitis [28]. However, treatment with MV restored the expression of the tight junction protein ZO-1, Claudin-1, and Occludin and reduced the permeability of the intestinal mucosa, thereby restoring intestinal barrier function.

ERS plays a crucial role in the pathogenesis and development of colitis [14, 29]. The enhanced and dysregulated ERS response in intestinal tissues of colitis patients is closely associated with various pathophysiological processes, including intestinal mucosal barrier damage, inflammatory cell infiltration, and apoptotic cell death [30, 31]. Therefore, recent studies have focused on modulating the ERS pathway to alleviate the onset and progression of colitis [32, 33]. Given the critical role of ERS in the pathogenesis of colitis, this study aimed to investigate the mechanism by which MV alleviates the symptoms of DSS-induced UC mice via the ERS pathway. Furthermore, we examined the effects of the ERS inhibitor 4-PBA and the ERS activator Tm on the treatment of UC [34, 35]. Our results revealed that the ERS pathway was activated in colitis mice. Treatment with MV reduced the major protein expression of the PERK-ATF4-eIF2α-CHOP pathway, thereby alleviating intestinal tissue damage. Furthermore, MV treatment led to reduced Caspase-12 protein expression and downregulation of the Bcl-2/Bax ratio, ultimately decreasing intestinal cell apoptosis. Treatment with the ERS inhibitor 4-PBA attenuated the intracellular ERS response and promoted normal protein folding, thereby enhancing the cytoprotective effect of MV. Conversely, treatment with the ERS activator Tm exacerbated ERS and reversed the therapeutic effect of MV.

Recently, nano-traditional Chinese medicine have been a promising strategy in consideration of their large specific surface area, strong targeting capability, good sustained-release effect [36]. The green synthesis of nanoparticles is a promising and environmentally friendly approach and the natural reagents such as herbs, bacteria, and fungi are used in the green synthesis of nanomaterials. Green nanomaterials is highly beneficial compared to traditional methods due to its low cost, and safety for the environment and human health [37]. Some studies have reported that quercetin supramolecular nanoribbons, nanoparticles from Platycladi Cacumen Carbonisata and the rhei radix rhizoma-based carbon dots ameliorates the UC in mice [38–40]. MV is expected to be developed into green nanomaterials for the treatment of UC in the future.

In conclusion, our study suggests that MV can alleviate the symptoms of DSS-induced UC mice by modulating the ERS pathway, reducing the apoptosis of intestinal cells, maintaining the integrity of the intestinal barrier, and alleviating the damage of intestinal tissues. These findings not only provide a reference for the development of the ERS pathway as a new target for the treatment of colitis but also present a novel idea and strategy for clinical treatment. Therefore, MV has the potential to be an effective drug for the treatment of colitis, with broad application prospects.
